# Habitual Flavonoid Intake from Fruit and Vegetables during Adolescence and Serum Lipid Levels in Early Adulthood: A Prospective Analysis

**DOI:** 10.3390/nu10040488

**Published:** 2018-04-14

**Authors:** Katharina J. Penczynski, Thomas Remer, Christian Herder, Hermann Kalhoff, Johanna Rienks, Daniel F. Markgraf, Michael Roden, Anette E. Buyken

**Affiliations:** 1 DONALD Study Dortmund, Department of Nutrition and Food Sciences (IEL), Nutritional Epidemiology, University of Bonn, 44225 Dortmund, Germany; katharina.penczynski@uni-paderborn.de (K.J.P.); anette.buyken@uni-paderborn.de (A.E.B.); 2 Department of Sports and Health, Institute of Nutrition, Consumption and Health, University of Paderborn, 33098 Paderborn, Germany; 3 Institute for Clinical Diabetology, German Diabetes Center, Leibniz Center for Diabetes Research at Heinrich-Heine University Düsseldorf, 40225 Düsseldorf, Germany; Christian.Herder@DDZ.UNI-DUESSELDORF.DE (C.H.); Daniel.Markgraf@DDZ.UNI-DUESSELDORF.DE (D.F.M.); Michael.Roden@DDZ.UNI-DUESSELDORF.DE (M.R.); 4 German Center for Diabetes Research, 85764 München-Neuherberg, Germany; 5 Pediatric Clinic Dortmund, 44317 Dortmund, Germany; Hermann.Kalhoff@klinikumdo.de; 6 Department of Nutrition and Food Sciences (IEL), Nutritional Epidemiology, University of Bonn, 53115 Bonn, Germany; jrienks@uni-bonn.de; 7 Division of Endocrinology and Diabetology, Medical Faculty, Heinrich-Heine University Düsseldorf, 40225 Düsseldorf, Germany

**Keywords:** 24-h urine, puberty, adulthood, biomarker of intake, blood lipid profile, flavonoids from fruit and vegetables, flavonoids from juices, high-density lipoprotein cholesterol, hippuric acid, low-density lipoprotein cholesterol

## Abstract

Flavonoids have been implicated in the prevention of cardiovascular diseases (CVD). In a prospective approach, we investigated whether habitual flavonoid intake from fruit, vegetables and juices (FlavFVJ) during adolescence is associated with adult levels of serum lipids, one of the main CVD risk factors. This analysis included healthy participants from the Dortmund Nutritional and Anthropometric Longitudinally Designed (DONALD) study, who had provided a fasting blood sample in adulthood (aged 18–39 years), data on FlavFVJ intake during adolescence (females: 9–15 years, males: 10–16 years)—estimated either from multiple 3-day weighed dietary records (*n* = 257), or from validated biomarker hippuric acid (uHA) excretion from multiple 24-h urine samples (*n* = 233)—together with information on relevant covariates. In multivariable linear regression analyses, a higher FlavFVJ intake during adolescence was independently associated with higher serum high-density lipoprotein cholesterol (HDL-C) levels among males (*P_trend_* = 0.038); however, the inclusion of adult waist circumference attenuated this association (*P_trend_* = 0.053). FlavFVJ was not associated with triglycerides (TG), total cholesterol (TC) or low-density lipoprotein cholesterol (LDL-C; all *P_trend_* ≥ 0.1), nor was uHA excretion with any serum lipid outcome among males (all *P_trend_* ≥ 0.5). Neither FlavFVJ intake nor uHA excretion was associated with serum lipids among women (all *P_trend_* ≥ 0.1). However, a higher flavonoid intake from fruit and vegetables was independently related to lower LDL-C levels (*P_trend_* = 0.021), while a higher intake from juices was associated with higher LDL-C levels (*P_trend_* = 0.016) among females. In conclusion, a higher flavonoid intake from fruit, vegetables and/or juices during adolescence may be linked to cholesterol levels in early adulthood in a sex- and food source-specific manner.

## 1. Introduction

Flavonoids continue to attract scientific interest owing to accumulating evidence regarding their promising health impacts [1,2,3]. These polyphenolic secondary plant constituents are classified into six main subclasses [3] of which flavonols, flavan-3-ols, flavanones, and anthocyanidins belong to those most widely consumed in Europe [4]. Among the health effects discussed for flavonoids, their antioxidant, anti-inflammatory and anti-thrombotic activities as well as their potential to improve blood lipid profiles, reduce the low-density lipoprotein (LDL) susceptibility to oxidation, reduce blood pressure, and improve endothelial function [1,2,5] are considered to be most relevant to the prevention of cardiovascular diseases (CVD). In line with these effects, a recent meta-analysis of prospective studies in adult populations supports the contribution of flavonoids to the prevention of CVD [6].

The reduction of increased serum lipid levels represents a main pillar of preventive efforts aimed at reducing the risk of major cardiovascular events [7]. It is widely recognized that effective long-term prevention of CVD in adulthood should start in youth [7,8]—a time frame in which the development of unfavourable blood lipid levels and its tracking into adulthood could be prevented by healthy lifestyle choices [8,9], possibly including flavonoid consumption.

To date, a possible relationship between flavonoid consumption during adolescence and blood lipid levels later in life remains uninvestigated. From a public health perspective, it is of particular interest whether the overall flavonoid intake from fruit, vegetables and juices (FlavFVJ)—i.e., the most important flavonoid sources among adolescents [10]—offers preventive potential. However, recent evidence suggesting that different fruit forms (whole fruit vs. clear juice) have different impacts specifically on LDL-cholesterol (LDL-C) levels [11], demands separate consideration of flavonoids from fruit and vegetables (FlavFV) and those from juices (FlavJ).

For optimal flavonoid intake estimation, the concomitant use of dietary instruments and validated intake biomarkers is recommended [12]. The excretion of urinary hippuric acid (uHA) in 24-h urine is one such surrogate biomarker of overall FlavFVJ intake, which we have recently validated against 3-day dietary records in healthy adolescents [13].

Taken together, current evidence regarding the preventive potential of flavonoids for CVD among adults as well as the fact that international health authorities unanimously call for preventive efforts starting early in life suggest that it is of interest to investigate whether flavonoid consumption during the critical period of adolescence offers a longer-term preventive potential for serum lipids in adulthood.

Hence, by applying both dietary and urinary biomarker approaches to estimate FlavFVJ intake, this study tests the hypothesis that habitual FlavFVJ intake during adolescence relates favourably to serum lipid levels in early adulthood.

## 2. Materials and Methods 

### 2.1. Study Population

The present analysis is based on data from the Dortmund Nutritional and Anthropometric Longitudinally Designed Study (DONALD Study) [14], an ongoing, open-cohort study conducted in Dortmund, Germany since 1985. Briefly, each year approximately 35–40 infants are newly recruited and detailed data on diet, growth, development, and metabolism are collected between infancy and adulthood. Examinations begin at the age of 3 months and are performed annually from childhood to adulthood. Since 2005, adult participants have been invited for subsequent examinations, including fasting blood withdrawal. The study was approved by the Ethics Committee of the University of Bonn (Germany; approval no. 098/06 and 071/04). Written parental consent and adult participants’ consent has been obtained for all examinations.

**Figure 1:**
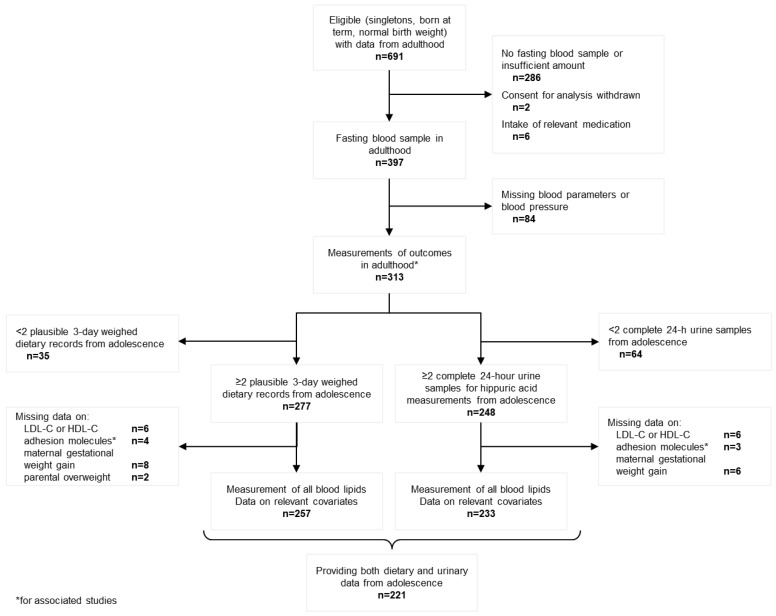
Participant flow diagram. HDL-C, high-density lipoprotein cholesterol; LDL-C, low-density lipoprotein cholesterol.

The ages of the children who were initially recruited into the DONALD study were quite variable. Due to the open-cohort design of the DONALD study, many children have not yet reached young adulthood or did not provide the data required for analysis. At the time of this analysis, 691 participants provided data from adulthood and fulfilled the following eligibility criteria: singletons, born at term (37 to <43 gestation weeks) with normal birth weight. Of these, 397 participants had provided a fasting blood sample in adulthood, of which 313 had measurements of relevant blood parameters used for associated studies [15,16]. To estimate habitual intake of FlavFVJ during adolescence (females: 9–15 years, males: 10–16 years), participants additionally had to have provided either at least two 3-day weighed dietary records (*n* = 277 after exclusion of 19 participants with ≥50% implausible dietary records) or at least two complete 24-h urine samples (*n* = 248) for the measurement of uHA excretion. Finally, after exclusion of missing lipid values (*n* = 6 missing LDL-C or high-density lipoprotein (HDL)-C values), anthropometric measurements from adolescence and adulthood and data on relevant covariates (*n* = 8 and *n* = 6 missing information on maternal gestational weight gain on the dietary level and urinary level, respectively, and *n* = 2 missing information on parental overweight on the dietary level), the resulting samples included 257 participants for dietary analysis and 233 participants for the urinary biomarker analysis (with *n* = 221 providing both dietary and urinary data, see Figure 1).

### 2.2. Dietary Assessment

Dietary intake was assessed by 3-day weighed dietary records, which included weighing and recording of all consumed foods and beverages as well as leftovers to the nearest g. Semi-quantitative recording was used if weighing was unfeasible. Nutrient intake was determined using the continuously updated in-house nutrient database, named LEBTAB [17].

As previously described [13], dietary FlavFVJ intake was estimated after assignment of flavonoid contents from U.S. Department of Agriculture (USDA) databases to the recorded food items. The impact of food processing on flavonoid contents was accounted for by retention and/or yield factors. Individual FlavFVJ-intake represents the sum of the flavones, flavonols, flavan-3-ol -monomers, flavanones, anthocyanidins and proanthocyanidins (i.e., flavan-3-ol-dimers to polymers). Subsequently, individual nutrient and FlavFVJ-intakes were averaged over the three recorded days. The participants’ habitual intakes were estimated by averaging data from all records collected during adolescence (2 to 7 records per person, mean = 6).

### 2.3. Urine Collection and Analysis

Each year, participants are requested to collect a 24-h urine sample. All micturitions from the 24-h sampling period are collected in provided Extran-cleaned (Extran, MA03, Merck, Darmstadt, Germany) preservative-free 1-L plastic containers and stored immediately at ≤−12 °C. After transport to the study centre, the samples are stored at ≤−20 °C until analysed.

uHA was measured photometrically, in triplicate, with the following modifications to the method from Tomokuni and Ogata [18]: (a) placement of the reagents in an ice bath to moderate the exothermic reaction; (b) prolongation of the reaction time of the urine with benzenesulfonyl chloride and pyridine to 60 min; (c) replacement of ethanol with methanol as the diluent; and (d) photometrical measurement at 436 nm. Photometric determination of uHA was performed on two spectrometers (Lambda 11UV/VIS Spectrometer, Perkin Elmer, Überlingen, Germany and Campspec UV/VIS Spectrophotometer M107, Spectronic Campspec Ltd., Leeds, UK) with conformity of measurements on both spectrometers ensured by Bland–Altman plots. The inter- and intra-assay precision for both spectrometers, expressed as coefficient of variation (CV), was 6.3% and 3.8%, respectively.

uHA excretions from all 24-h urine samples collected during adolescence (2 to 5 samples per person, mean = 4.5) were averaged to reflect habitually ingested flavonoids.

### 2.4. Blood Sampling and Analysis

Venous blood samples were drawn after an overnight fast, centrifuged at 4 °C within 15 min and stored at −80 °C. Plasma triglycerides (TG) were measured at the German Diabetes Center with the Roche/Hitachi Cobas c311 analyser (Roche diagnostics, Mannheim, Germany). Serum concentrations of total cholesterol (TC), LDL-C and HDL-C were determined at the Paediatric Clinic Dortmund using the Advia 1650-Chemistry System analyser (Siemens Healthcare Diagnostics, Eschborn, Germany).

### 2.5. Anthropometric Measurements and Assessment of Additional Data

Standing height was measured to the nearest 0.1 cm (digital stadiometer: Harpenden Ltd., Crymych, UK) and body weight to the nearest 0.1 kg (electronic scale: Seca 753E, Seca Weighing and Measuring Systems, Hamburg, Germany). From these measures, the participants’ body surface area (BSA), body mass index standard deviation (BMI SD) scores (sex- and age-specifically standardized according to German references [19]) were calculated. Overweight during puberty was defined according to the International Obesity Task Force BMI cutoffs [20]. Waist circumference was measured at the midpoint between the lower rib and iliac crest to the nearest 0.1 cm.

On their child’s admission to the DONALD study, parents were anthropometrically and medically examined and interviewed about the child’s early life factors, their disease history and socioeconomic status.

### 2.6. Statistical Analysis

The characteristics of the study population are presented as means ± SD or medians (25th, 75th percentiles) for continuous variables and as percentages for categorical variables.

To achieve normal distributions for the outcome and exposure variables, we used log_e_ and square root transformations. Individual outliers were winsorized (<1% of the data for HDL-C). Before calculating the individual means from available records or urine during adolescence, dietary variables were energy-adjusted by the residual method and standardized by age group and sex (mean = 0, SD = 1) to account for age- and sex-dependent intake differences. Analogously, uHA was adjusted for BSA (standardized by age group and sex; mean = 0, SD = 1), which, due to its close link with individual body size-dependent glomerular filtration rates [21] codetermines uHA and may serve as a proxy measure for energy requirements.

Prospective associations between FlavFVJ or uHA during adolescence and serum lipids in early adulthood were analysed by multivariable linear regression models, using the transformed variables, as explained above. Formal interaction analyses indicated sex interactions for HDL-C, LDL-C and TC on the dietary level (*P*_interaction_ = 0.01 to 0.07); for comparability reasons, all analyses were sex-stratified.

Initial regression models (model A) included the predictors (as age- and sex-specific studentized residuals as explained above) and age at blood withdrawal. Adjusted models (model B) were constructed by individual examination of potential influencing covariates and hierarchical inclusion [22] of those which substantially modified the predictor–outcome associations (≥10%) or significantly predicted the outcome. Potential confounding covariates considered in the hierarchical approach were (1) early life factors (birth weight (g), gestational age (week), maternal age at birth (year), gestational weight gain (kg) and exclusive breastfeeding for >2 weeks (yes/no)); (2) socioeconomic factors and parental health status (smokers in the household (yes/no), paternal school education ≥12 years (yes/no) and presence of an overweight parent(BMI ≥25 kg/m^2^; yes/no)) and (3) predictor-specific adolescent data (pubertal BMI-SD score and energy-adjusted dietary intake (protein, total and saturated fat, carbohydrate, added sugar and fibre (total, soluble and insoluble; as age- and sex-specific studentized residuals) in models with the dietary predictor, FlavFVJ, and pubertal BSA (age- and sex-specifically studentized) in models with the urinary predictor uHA). In conditional models, we additionally included adult waist circumference to investigate whether observed associations were partly attributable to body composition in adulthood. Results from regression analyses are presented as adjusted least-square means (95% confidence interval (CI)) by tertiles of the respective predictor while *P*-values stem from models using the predictors as continuous variables.

In addition, the separate association of flavonoids from fruit and vegetables (excluding juices; FlavFV) vs. those from juices (FlavJ) on LDL-C was investigated. Furthermore, sensitivity analyses were conducted in subsamples of participants, providing data on (a) adult FlavFVJ intake (*n* = 229 in dietary sample); (b) adult alcohol consumption (*n* = 229 in dietary sample, *n* = 204 in urinary sample); (c) adult smoking status (*n* = 254 in dietary sample, *n* = 229 in urinary sample) and (d) adult physical activity (*n* = 256 in dietary sample, *n* = 232 in urinary sample).

The SAS statistical software package version 9.2 (SAS Institute Inc., Cary, NC, USA) was used for all statistical analyses. The significance level was set at *P* < 0.05. 

## 3. Results

The characteristics of our samples are shown in Table 1. While the median absolute intakes of FlavFVJ in adolescence were 129 and 130 mg/day in males and females, respectively (Table 1), the median consumption of FlavFVJ per megajoule (MJ) amounted to 14.4 and 18.3 mg/MJ in males and females, respectively. 

Among males, a higher habitual FlavFVJ intake was independently related to higher HDL-C values only (*P_trend_* = 0.038; Table 2, model B), which was not corroborated by the biomarker uHA. Additional inclusion of adult waist circumference attenuated this association to a trend (*P_trend_* = 0.053; Table 2, conditional model). Among females, neither predictor was associated with any lipid outcome (Table 3).

**Figure 2:**
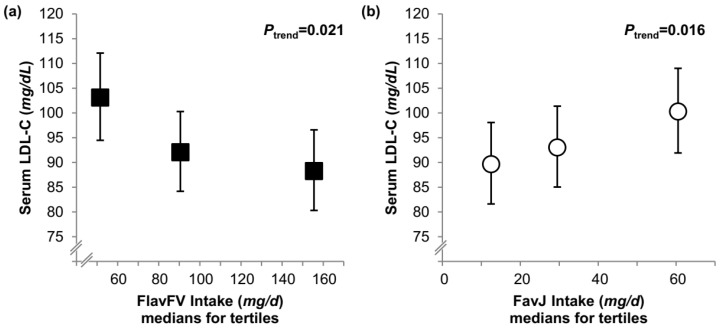
Serum levels of LDL-C in early adulthood by tertiles of dietary flavonoid intake from fruit and vegetables (FlavFV, panel (**a**), ■) and flavonoid intake from juices (FlavJ, panel (**b**), ○) during adolescence among females (*n* = 134). Data are geometric means and 95% CI adjusted for age at blood withdrawal, maternal gestational weight gain, intake of added sugar (residuals on FlavFVJ intake), energy (residuals) and vice versa adjustment for FlavJ (**a**) or FlavFV (**b**) intake during adolescence.

Separate analysis of the relationship of FlavFV vs. FlavJ with LDL-C (which can only be performed on the dietary level) revealed a diverging association solely among females—higher intakes of FlavFV were associated with lower LDL-C, while higher FlavJ intakes were related to higher LDL-C (*P_trend_* = 0.021 and *P_trend_* = 0.016, respectively; Figure 2).

Sensitivity analyses in subsamples of participants with additional data from adulthood mainly identified adult alcohol consumption as being potentially relevant; the inclusion of this strengthened the association between FlavFVJ and HDL-C among males (*P_trend_* = 0.075 in model B and *P_trend_* = 0.035 in model B + adult alcohol intake, *n* = 113).

## 4. Discussion

This prospective study suggests an association between higher habitual flavonoid intake from fruit, vegetables and/or juices during adolescence and serum lipid levels in adulthood. However, this appears to depend on sex and food source. Males consuming more flavonoids from fruit, vegetables and juices had higher HDL-C levels. Among females, LDL-C values were differentially related to different sources of flavonoids, with an inverse association for flavonoids from fruit and vegetables and a direct association with those from juices. Those associations emerged solely on the dietary level.

There have been several previous observational studies that have investigated how flavonoid or polyphenol intake relates to blood lipids, but, to the best of our knowledge, only one of those used a prospective design [23]. In that study, which involved five-year follow-up of adults at high cardiovascular risk, higher urinary excretion of total polyphenols was associated with lower TG, however not with TC, HDL-C or LDL-C levels [23]. All other observational studies were cross-sectional studies in adults and yielded contradictory results regarding the association between total flavonoid intake and HDL-C [24,25,26,27,28] or LDL-C [25,26,27]. Whereas a higher total flavonoid intake was mostly related to lower TG levels [24,25,26,27,28], no associations were found with TC [26,27]. Evidence for flavonoid subclasses is also inconsistent [26,27,29,30,31].

Similarly, meta-analyses of randomized controlled trials (RCT) on the effect of various flavonoid subclasses [32,33,34,35] or fruit, vegetables or juices [35,36,37,38] have yielded inconclusive results. Some of these meta-analyses are in accordance with our results, as they report that interventions with anthocyanins, flavonols or berries resulted in decreased LDL-C [32,33,38] and/or increased HDL-C levels [32,33]. Taken together, the overall current evidence is partly inconclusive and so far, does not allow for unequivocal judgment on the relevance of flavonoids for serum blood lipids.

The reason for the sex specificity of our results is unclear. Since sex and gender differences are apparent in lipid metabolism [39,40] as well as in pharmacokinetics [41], it is conceivable that sexual dimorphism could extend to the actions of flavonoids on blood lipid profiles. Indeed, two cross-sectional studies have also reported sex-specific associations of polyphenols or flavonoids with serum lipid levels [26,28]; however, these sex specific results neither resemble one another, nor those seen in our study. To the best of our knowledge, mechanistic evidence of a possible differential effect of flavonoids on blood lipids between males and females is lacking. With such inconsistency between the reported sex specificities and missing mechanistic explanations, additional studies are needed to further investigate the possible sex differences regarding the impact of flavonoids on blood lipids. Except for a possible but unknown mechanistic basis, reasons for our sex specific results may lie in differing relative flavonoid intakes and serum lipid concentrations among males and females.

The differential association of FlavFV vs. FlavJ with LDL-C found in our study is in line with an RCT comparing the effect of whole apples vs. clear apple juice on LDL-C concentrations [11]. Ravn-Haren et al. concluded that this differential effect is attributable to the differing pectin content rather than the polyphenolic content of the apple products [11]. In our study, however, additional adjustment for fibre intake (total, soluble or insoluble fibre) did not interfere with the differential flavonoid-LDL-C associations; similarly, dietary fructose and dietary glucose intake appeared to be irrelevant. Still, there is no mechanistic reason to believe that flavonoids from whole fruit and vegetables should act in another way than those from juices. As the reason for the differential association remains unresolved, a potential contribution of unmeasured confounding to this finding needs to be considered.

It could be argued that the flavonoid–lipid associations result from differential overreporting or imprecise flavonoid databases as they were only discernible on the dietary level. However, source-specific associations with LDL could not be addressed on the biomarker level as uHA does not allow differentiation between flavonoid sources. In addition, two points support the interpretation of our observations as a “real” effect: Firstly, mechanistic in vitro, animal, and human studies have demonstrated multiple biologically plausible mechanisms involved in cholesterol synthesis, reverse cholesterol transport and cholesterol clearance [1,42] by which flavonoids may ultimately impact on serum lipid levels. Secondly, our observed effect sizes agree with those reported in the literature. In our study, the highest vs. the lowest tertile of FlavFVJ intake was related to 4 mg/dL (8%) higher HDL-C levels among males, while the highest vs. the lowest tertile of FlavFV intake (but not FlavFVJ) translated into 15 mg/dL (14%) lower LDL-C levels among females. With that, our effect sizes are moderately clinically relevant and lie within the reported ranges: HDL-C increases ranged from 0.5% for a 100% raise in total flavonoid intake [27] to 13% higher HDL-C levels in the highest vs. the lowest tertile of anthocyanin intake [26] in cross-sectional studies, and from 2 to 6 mg/dL in interventions with anthocyanins (dosages: 90–320 mg/day) or flavonols (dosages: 16–1200 mg/day) in RCTs [32,33]. The effect size for LDL-C was clinically irrelevant in the cross-sectional study [25], yet ranged from a 5 to a 22 mg/dL decrease in interventions with anthocyanins or flavonols (dosages as above) [32,33].

Finally, our longer-term associations could reflect either (i) the downstream effects of a benefit from flavonoids on body composition in adulthood; (ii) a tracking in dietary behaviour from adolescence to early adulthood or (iii) a shorter-term impact of flavonoids on serum lipids in adolescence and tracking of this beneficial lipid profile into early adulthood. Our additional analyses did not support the two first mentioned concerns since (i) the consideration of adult waist circumference in conditional models had no influence on the results in relation to LDL-C (data not shown) and marginally attenuated the association with HDL-C among males as reflected by nearly unchanged least squares means and 95% CIs (model B vs. conditional model) and (ii) the additional consideration of adult intake levels did not considerably affect the results. Hence, the observed associations may indeed reflect a long-term setting of lipid metabolism, which is still discernible several years later. However, the possibility of (iii) the tracking of a benefit on the serum lipid profile from adolescence to early adulthood cannot be elucidated due to the fact that blood samples were not available from adolescence. Irrespective of this unknown contribution, we contend that the clinical relevance of our association is moderate in comparison to concurrent influences (e.g., current weight or alcohol intake).

Our study is mainly limited by the specific sources of measurement error intrinsic to both methods of FlavFVJ intake estimation—i.e., the reliance on self-reports and incomplete databases for dietary records [12] and the interference of other precursors of uHA, such as phenolic acids, with the validity of uHA as a biomarker of FlavFVJ [13]. Another limitation is the single measurement of serum lipid concentrations and—as outlined above—the lack of serum lipid measurements from adolescence. The study is further limited by the fact that our sample is not representative of the total population and due to the lack of uHA measurements from early adulthood for sensitivity analyses on the urinary level.

The strengths of our study include its prospective design, the estimation of habitual FlavFVJ from repeated exposure assessments, and the use of a validated biomarker of overall FlavFVJ intake. Furthermore, exposure estimation on both the dietary and the biomarker levels, using two specifically detailed and accurate methods, provides an insight into the consistency of results.

In conclusion, our data suggest a possible sex- and food source-specific benefit of a higher habitual flavonoid intake from fruit, vegetables and/or juices during adolescence on the serum lipid profile in early adulthood.

## Figures and Tables

**Table 1:** Characteristics of the participants in adolescence (males: 10–16 years, females: 9–15 years) and early adulthood: anthropometry, dietary and urinary data, serum lipid levels, early life and socioeconomic factors ^1^.

	Dietary Sample (*n* = 257)		Urinary Sample (*n* = 233)	
	Males (*n* = 123)	Females (*n* = 134)	Males (*n* = 115)	Females (*n* = 118)
**Data from adolescence**				
Age (years)	13.0 (12.9, 13.0)	12.0 (11.9, 12.0)	12.8 (12.2, 13.3)	11.8 (11.4, 12.4)
**Anthropometry, dietary and urinary data**				
BMI-SD score	−0.19 ± 0.77	−0.24 ± 0.93	−0.09 ± 0.84	−0.19 ± 0.93
BMI (kg/m^2^)	18.8 (17.6, 20.2)	17.7 (16.5, 20.1)	19.3 (17.5, 20.7)	18.0 (16.7, 20.5)
BSA (m^2^)	1.5 (1.4, 1.6)	1.4 (1.3, 1.5)	1.5 (1.4, 1.6)	1.4 (1.3, 1.5)
Overweight (%) ^2^	22.0	21.6	28.7	22.9
Total energy (MJ/day)	8.9 (8.1, 10.2)	7.1 (6.5, 8.0)		
Fat (%en)	35.4 ± 3.9	36.0 ± 4.0		
SFA (%en)	15.5 ± 2.1	15.9 ± 2.1		
Protein (%en)	13.1 ± 1.3	12.9 ± 1.7		
Carbohydrate (%en)	51.4 ± 4.0	51.0 ± 4.5		
Fibre (g/MJ)	2.33 (2.05, 2.76)	2.52 (2.18, 2.82)		
FVJ (g/day)	465 (355, 612)	423 (314, 534)		
FlavFVJ (mg/day)	129 (86, 189)	130 (88, 173)		
FlavFVJ (mg/MJ)	14.4 (10.1, 20.5)	18.3 (12.8, 24.4)		
FlavFV (mg/day)	80 (51, 133)	90 (59, 136)		
FlavJ (mg/day)	40 (23, 64)	29 (19, 49)		
Urinary hippuric acid (mmol/24 h)			3.0 (2.6, 3.6)	2.6 (2.3, 3.2)
E**arly life and socioeconomic factors**				
Birth weight (g)	3500 (3170, 3840)	3428 (3100, 3750)	3550 (3200, 3850)	3405 (3100, 3730)
Gestational age (week) ^3^	40 (39, 41)	40 (40, 41)	40 (39, 41)	40 (40, 41)
Maternal gestational weight gain (kg) ^3^	12 (9, 14)	12 (10, 15)	12 (9, 15)	12 (10, 15)
Maternal age at birth (year)	30.7 (28.1, 33.7)	29.8 (27.7, 32.7)	30.6 (28.1, 33.7)	29.9 (27.7, 33.2)
Smokers in the household (%)	24.4	35.8	27.0	34.7
Paternal high education (%) ^3,4^	64.5	55.6	61.7	54.0
Overweight parent (%) ^3,5^	73.2	67.2	76.3	70.3
**Data from early adulthood**				
Age (years)	20.9 (18.1, 23.2)	21.7 (18.1, 24.9)	19.6 (18.1, 23.0)	21.3 (18.1, 24.5)
**Anthropometry, dietary and lifestyle data**				
BMI (kg/m^2^)	22.8 (21.1, 25.6)	21.9 (20.2, 24.1)	23.1 (21.1, 26.2)	21.9 (20.3, 24.3)
Waist circumference (cm)	79.2 (75.6, 87.3)	72.0 (67.8, 76.8)	79.7 (75.7, 87.6)	72.1 (68.0, 77.0)
Total energy (MJ/day) ^3^	10.6 (9.3, 12.5)	7.9 (6.6, 8.9)		
FVJ (g/day) ^3^	423 (247, 712)	472 (304, 627)		
FlavFVJ (mg/day) ^3^	99 (39, 174)	114 (71, 175)		
Alcohol (g/day) ^3^	1.2 (0.1, 12.3)	0.2 (0.1, 2.6)		
Current smoker (%) ^3^	26.2	24.2	29.8	21.7
**Serum lipid levels**				
TG (mg/dL)	82 (68, 123)	97 (73, 120)	83 (68, 124)	94 (73, 120)
TC (mg/dL)	157 (137, 188)	178 (155, 203)	157 (139, 188)	179 (157, 204)
LDL-C (mg/dL)	91 (73, 111)	95 (77, 112)	90 (73, 109)	94 (77, 113)
HDL-C (mg/dL)	50 (43, 59)	65 (54, 77)	50 (42, 59)	66 (54, 77)

^1^ Values are means ± SD, medians (25th, 75th percentiles) or relative frequencies. BSA, body surface area; DONALD, Dortmund Nutritional and Anthropometric Longitudinally Designed; FlavFV, dietary flavonoids from fruit and vegetables excluding juices; FlavFVJ, dietary flavonoids from fruit and vegetables including juices; FlavJ, dietary flavonoids from juices; FVJ, fruit and vegetables including juices; HDL-C, high-density lipoprotein cholesterol; LDL-C, low-density lipoprotein cholesterol; MJ, megajoule; SFA, saturated fatty acids; TC, total cholesterol; TG, triglycerides; ^2^ defined according to age- and sex-specific cut points of the International Obesity Task Force (Cole et al., 2000 [20]); ^3^ reduced sample sizes in the dietary sample occurred for paternal high education (*n* = 247), dietary data from adulthood (*n* = 229) and current smokers (*n* = 254); and in the urinary sample, for overweight parent (*n* = 232), paternal high education (*n* = 228), current smoker (*n* = 229); ^4^ defined as school education ≥12 years; ^5^ Defined as BMI ≥25 kg/m^2^ in either of both parents at any interview time point.

**Table 2:** Prospective associations of dietary flavonoid intake from fruit and vegetables including juices (FlavFVJ) and urinary hippuric acid (uHA) excretion during adolescence with serum lipid levels in early adulthood among males ^1^.

	Tertiles of FlavFVJ Intake during Adolescence (*n* = 123)				Tertiles of uHA Excretion during Adolescence (*n* = 115)			
Outcomes	T1	T2	T3	*P*_trend_	T1	T2	T3	*P*_trend_
FlavFVJ (mg/day)^2^	68 (55, 86)	128 (115, 148)	206 (187, 235)					
uHA (mmol/24 h)^2^					2.3 (2.0, 2.6)	3.0 (2.7, 3.3)	4.0 (3.4, 4.7)	
TG (mg/dL)								
Model A	87 (76, 99)	89 (78, 102)	91 (80, 105)	0.9	92 (80,106)	91 (79, 105)	87 (76, 101)	0.7
Model B	89 (78, 102)	90 (78, 103)	89 (78, 102)	0.8	94 (82, 109)	90 (79, 104)	86 (75, 99)	0.7
Conditional model	88 (78, 101)	88 (77, 100)	91 (80, 105)	>0.9	93 (81, 108)	90 (79, 103)	87 (76, 100)	0.7
TC (mg/dL)								
Model A	153 (143, 163)	166 (156, 176)	167 (157, 178)	0.099	163 (153, 174)	162 (152, 172)	165 (154, 175)	0.5
Model B	155 (145, 165)	165 (154, 175)	167 (157, 177)	0.1	164 (154, 175)	162 (151, 172)	164 (154, 175)	0.5
Conditional model	155 (145, 165)	163 (153, 174)	168 (158, 178)	0.1	164 (154, 174)	161 (151, 171)	165 (155, 175)	0.4
HDL-C (mg/dL)								
Model A	48 (45, 52)	52 (48, 55)	51 (48, 55)	0.053	51 (48, 55)	49 (45, 52)	53 (49, 56)	0.4
Model B	48 (45, 51)	52 (48, 55)	52 (48, 55)	0.038	52 (48, 55)	49 (45, 53)	52 (48, 56)	0.6
Conditional model	48 (45, 51)	52 (49, 56)	51 (48, 55)	0.053	52 (48, 55)	49 (46, 53)	52 (48, 56)	0.6
LDL-C (mg/dL)								
Model A	85 (77, 93)	92 (84, 100)	94 (86, 102)	0.3	90 (82, 98)	92 (84, 100)	89 (81, 97)	0.6
Model B	87 (80, 95)	89 (82, 97)	93 (86, 101)	0.4	90 (82, 99)	91 (83, 100)	89 (81, 97)	0.5
Conditional model	87 (80, 95)	88 (81, 96)	95 (87, 103)	0.3	90 (82, 98)	91 (83, 99)	90 (82, 98)	0.4

^1^ Values are adjusted least-squares means (95% CIs) unless otherwise indicated. Linear trends (*P*_trend_) were obtained in linear regression models with FlavFVJ or uHA as a continuous variable. Model A with the predictor, FlavFVJ, adjusted for adult age at blood withdrawal and energy intake (age- and sex-standardized residuals). Model A with the predictor, uHA, adjusted for adult age at blood withdrawal, and BSA (age- and sex-standardized residuals). Model B with the predictor, FlavFVJ, additionally adjusted for the presence of an overweight parent, adolescent BMI-SD score and fibre intake (residuals on FVJ intake). Model B with the predictor, uHA, additionally adjusted for maternal gestational weight gain and adolescent BSA. Conditional model, additionally adjusted for adult waist circumference. Transformations of variables for analysis: log_e_ for uHA, log_e_ log_e_ for TG, square root for FlavFVJ, LDL-C and HDL-C. FlavFVJ, dietary flavonoids from fruit and vegetables including juices; HDL-C, high-density lipoprotein cholesterol; LDL-C, low-density lipoprotein cholesterol; TC, total cholesterol; TG, triglycerides; uHA, urinary hippuric acid. ^2^ values are unadjusted medians (25th, 75th percentiles).

**Table 3:** Prospective associations of dietary flavonoid intake from fruit and vegetables including juices (FlavFVJ) and urinary hippuric acid (uHA) excretion during adolescence with serum lipid levels in early adulthood among females ^1^.

	Tertiles of FlavFVJ Intake during Adolescence (*n* = 134)				Tertiles of uHA Excretion during Adolescence (*n* = 118)			
Outcomes	T1	T2	T3	*P*_trend_	T1	T2	T3	*P*_trend_
FlavFVJ (mg/day)^2^	74 (60, 87)	131 (111, 144)	199 (173, 228)					
uHA (mmol/24 h)^2^					2.2 (1.9, 2.5)	2.6 (2.4, 2.8)	3.4 (2.8, 4.1)	
TG (mg/dL)								
Model A	95 (86, 106)	91 (82, 101)	94 (84, 104)	0.8	93 (83, 104)	92 (82, 103)	91 (81, 102)	0.9
Model B	93 (84, 104)	91 (82, 101)	96 (86, 107)	0.7	93 (84, 105)	92 (82, 103)	91 (81, 101)	>0.9
Conditional model	93 (84, 104)	90 (82, 101)	96 (86, 107)	0.8	93 (83, 104)	91 (82, 102)	91 (82, 103)	0.9
TC (mg/dL)								
Model A	189 (179, 199)	175 (165, 186)	174 (164, 184)	0.071	185 (174, 196)	180 (169, 192)	178 (167, 189)	0.2
Model B	188 (178, 199)	176 (166, 186)	174 (163, 184)	0.1	185 (173, 197)	180 (169, 192)	178 (167, 190)	0.2
Conditional model	188 (178, 199)	176 (166, 186)	174 (163, 184)	0.1	184 (173, 196)	180 (168, 191)	179 (168, 191)	0.3
HDL-C (mg/dL)								
Model A	68 (63, 73)	63 (59, 68)	63 (59, 68)	0.2	67 (62, 73)	63 (58, 69)	65 (60, 70)	0.5
Model B	69 (64, 74)	63 (58, 68)	62 (58, 67)	0.1	68 (62, 73)	63 (58, 69)	65 (59, 70)	0.4
Conditional model	69 (64, 74)	63 (59, 68)	62 (58, 67)	0.1	68 (63, 73)	64 (59, 69)	64 (59, 69)	0.3
LDL-C (mg/dL)								
Model A	101 (92, 109)	89 (81, 98)	93 (85, 102)	0.2	96 (87, 106)	95 (86, 105)	95 (86, 105)	0.6
Model B	100 (91, 109)	90 (82, 99)	93 (85, 102)	0.3	96 (87, 106)	95 (86, 105)	96 (87, 106)	0.7
Conditional model	100 (91, 109)	90 (82, 99)	93 (85, 102)	0.3	95 (86, 105)	94 (86, 104)	97 (88, 107)	0.8

^1^ Values are adjusted least-squares means (95% CIs) unless otherwise indicated. Linear trends (*P*_trend_) were obtained with linear regression models with FlavFVJ or uHA as a continuous variable. Model A with the predictor, FlavFVJ, adjusted for adult age at blood withdrawal, and energy intake (age- and sex-standardized residuals). Model A with the predictor, uHA, adjusted for adult age at blood withdrawal, and BSA (age- and sex-standardized residuals). Model B with the predictor, FlavFVJ, additionally adjusted for maternal gestational weight gain and adolescent added sugar intake (residuals on FlavFVJ intake). Model B with the predictor, uHA, additionally adjusted for full breastfeeding, smokers in the household and adolescent BSA. Conditional model, additionally adjusted for adult waist circumference. Transformations of variables for analysis: log_e_ for uHA, log_e_ log_e_ for TG, square root for FlavFVJ, LDL-C and HDL-C. FlavFVJ, dietary flavonoids from fruit and vegetables including juices; HDL-C, high-density lipoprotein cholesterol; LDL-C, low-density lipoprotein cholesterol; TC, total cholesterol; TG, triglycerides; uHA, urinary hippuric acid; ^2^ values are unadjusted medians (25th, 75th percentiles).
